# Genome-wide association study of cocoon-producing traits in four Henan silkworm strains revealed new candidate genes

**DOI:** 10.3389/fgene.2025.1580806

**Published:** 2025-06-10

**Authors:** Xingya Song, Yingxian Lyu, Yuqiao Zhang, Wenjia Ding, Yongzhen Huang, Shang Yang

**Affiliations:** ^1^ Henan Academy of Sericulture Science, Zhengzhou, Henan, China; ^2^ College of Animal Science and Technology, Northwest Agriculture and Forestry University, Yangling, Shaanxi, China; ^3^ Henan Agricultural Society, Zhengzhou, Henan, China

**Keywords:** genome-wide association study, cocoon-producing traits, Henan silkworm strains, candidate genes, KWMTBOMO02490

## Abstract

**Background:**

The silkworm (*Bombyx mori*), has been farmed in China for over 5,000 years, and holds significant economic value. Recent genomic advances have deepened our understanding of silk production mechanisms, enabling the development of improved silkworm breeds. Further research is needed to identify effective molecular markers linked to silk production traits, as this will enhance genetic improvement efforts.

**Results:**

In the genome-wide association analysis study, we identified 40 significant single nucleotide polymorphisms (SNPs) and 28 candidate genes which were related to cocoon shell weight, cocoon width and whole cocoon weight in four silkworm strains in Henan. Through linkage imbalance block analysis, we found that the *KWMTBOMO02490* (ATPase inhibitor-like protein) and *KWMTBOMO12678* (OTU domain-containing protein 7B) were strongly selected. By using online databases, we found that the *KWMTBOMO02490* gene was highly expressed in the posterior silk glands and may play an important role in the process of silk production.

**Conclusion:**

The candidate genes in this study may affect the production and health traits of silkworms, and may provide an important reference for silkworm breeding.

## 1 Background

The silkworm (*Bombyx mori*) is one of the insects with high economic value. Its main product, silk, played an important role in the development of the “Silk Road”, the first large-scale commercial exchange between the East and the West in the world history. According to historical records, China’s sericulture has an ancient origin of 5,000 years. Silkworms likely originated in China as trimoulting lines and diversified into local strains through independent spreads along the Silk Road (Xiang et al., 2018). China’s long history of sericulture established its industry in an important position in the world.

Silk is the most important product of silkworms, and it is very important to unravel the genetic mechanism of silk-production. In recent years, China has accelerated the research on silkworm genomics, which is now at the forefront of the world. In 2003, two teams independently performed whole genome sequencing (WGS) of the domesticated silkworm genome and published the draft sequence for the silkworm genome (Xia et al., 2004; Mita et al., 2004). In 2009, Southwest University conducted whole genome resequencing of 29 strains of domesticated silkworms and 11 strains of wild silkworms, constructed a high-precision genetic variation map of the nuclear genome of silkworms,and found that the domestic silkworm had successfully differentiated from the wild silkworm after 5,000 years by phylogenetic analysis (Xia et al., 2009). Genome-based mapping studies have identified many important genes associated with mutations in body color (Futahashi et al., 2008), cocoon color (Sakudoh et al., 2007), development (Lu Z. et al., 2019), disease resistance (Yang et al., 2017), and other traits. A study on the domestication from wild silkworms to domestic silkworm has for the first time applied genome-wide association analysis (GWAS) to the mapping of important traits (cocoon color, blood color, disease resistance et al.) in the chromosomes of *B. mori*, confirming the feasibility and scientific validity of the methodology (Xiang et al., 2018). In 2022, the genome analysis of large-scale silkworm germplasm resources (“Thousand Silkworm Genome”) has been completed resulting in 87 the drawing of a super pan-genome map of silkworms (Tong et al., 2022). It provides a large amount of genomic information for the identification of important silkworm genes and the selection and breeding of silkworms. For silk production traits, we need to find more effective molecular markers to provide a scientific basis for genetic improvement of silkworms.

In this study, phenotypic statistics and whole-genome sequencing were performed on four silkworm strains in Henan Province with the aim of screening candidate genes for silkworm cocoon traits to help understand the genetic basis of silkworm production.

## 2 Materials and methods

### 2.1 Samples and phenotype collection

A total of 120 silkworms of the four strains were provided by the Henan Academy of Sericulture, and all of them were maintained under identical husbandry and management conditions. The four strains are Cuihua (CH, n = 30), Chunhui (CHH, n = 30), Chunyun A (CHY, n = 30), and Huayu A (HY, n = 30). Thirty individuals were selected from each strain, and the traits including whole cocoon weight (WCW), cocoon shell weight (CSW), cocoon length (CL), and cocoon width (CW) were measured. We used electronic analytical balances to measure WCW and CSW, and vernier calipers to measure CL and CW. Genomic DNA of each individual was extracted by a standard phenol-chloroform extraction method.

### 2.2 Sequencing, mapping and SNP calling

Genomic sequencing of these samples was performed by Huazhi Rice Bio-Tech Company (Changsha, China) using DNBSEQ-T7. Reads of 300 bp of qualified DNA samples were used to construct DNA libraries. Clean data were obtained by FASTP (Chen et al., 2018) after removing low quality reads, adaptors, and reads with multiple N bases. Read quality was assessed through FastQC (v0.11.9). Subsequently, clean reads were mapped to the silkworm reference genome (Kawamoto et al., 2019) using Sentieon (Freed et al., 2017). QualiMap (v2.2.1) (Okonechnikov et al., 2016) was used to evaluate sequencing alignment data. Each sample was detected for SNPs by Sentieon (Freed et al., 2017),and then these samples are combined into a variant call format (VCF) file and filtered with parameters including “QD < 2.0, FS > 60.0, MQ < 40.0, SOR >3.0, MQRankSum < −12.5, ReadPosRankSum < −8.0”. Then, we used ANNOVAR (Wang et al., 2010) to annotate these SNPs.

The quality of the resulting SNP genotype data was improved by the removal of SNPs with detection rate <95%, and criteria such as minor allele frequency (MAF) < 0.05, and a Hardy-Weinberg Equilibrium (HWE) *P*-value < 1e–4. Finally, we used PLINK (version 1.90) (Purcell et al., 2007) to filter the SNP data by “--indep-pairwise 500 50 0.5” parameters to remove the SNPs which have high linkage disequilibrium (LD) values. This procedure can reduce the amount of non-essential computation.

### 2.3 Population structure analysis

Since the silkworms involved in this study came from different populations, distinct subgroups may exist among these silkworms. We used principle component analysis (PCA) to explore the stratification of the populations by smartPCA (Patterson et al., 2006), and PopLDdecay (Zhang et al., 2019) was used to explore the LD degree.

### 2.4 GWAS analysis

We used the Linear Mixed Model (LMM) model of GEMMA (v0.98.5) (Zhou and Stephens, 2012) software for GWAS analysis. If points in quantile-quantile (Q-Q) plots are off the diagonal (especially at the left end), population stratification or false positives in the model are suggested. λ corresponds to the genome inflation factor, with λ closer to 1 indicating a better model fit. Each point in the Manhattan plot was used to display SNPs, in which values in the vertical coordinate represent the strength of the association of the SNP with the phenotype. In order to reduce the effect of population stratification on GWAS, we used the first three principal components as covariates. The specific statistical model of GWAS is as follows:

y=SNP+COV+Kin+e

With ***y*** the phenotype value, *Kin* the kinship matrix, *SNP* the SNP marker effect, *COV* is the covariate, and *e* is the residual effect. The significance threshold (the red dotted lines in Manhattan plots) was calculated using the *Bonferroni* correction method (0.05/175,704 = 2.845e-7).

### 2.5 Gene annotation and LD blocks plotting

Genes within a 10 kb region centered on the significant SNPs were identified using BEDTools (Quinlan and Hall, 2010). The 10 kb range was selected based on the gene annotation region in the previous study (5 kb each upstream and downstream) (Lu et al., 2023). We used LDBlockshow (Dong et al., 2021) to map LD blocks in the 10 kb upstream and downstream range of genes. R^2^ represents the degree of association, and the closer the value is to 1, the redder the color in the LD blocks and the higher the degree of linkage disequilibrium.

## 3 Result

### 3.1 Phenotypic values and sequencing data

**FIGURE 1:**
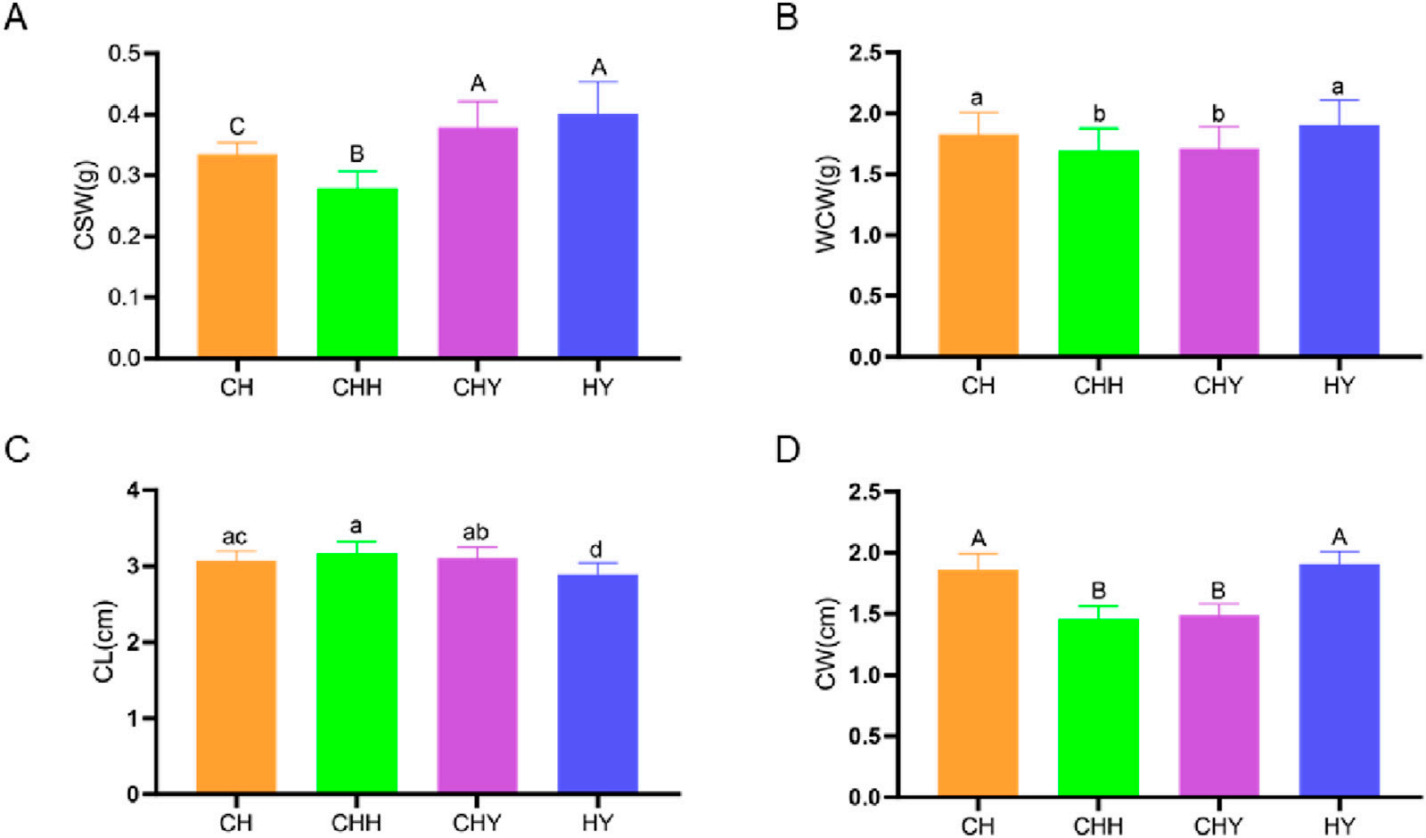
Histograms of phenotype values for four cocooning traits in four breeds. **(A)** Cocoon shell weight (CSW). **(B)** Whole cocoon weight (WCW). **(C)** Cocoon length (CL). **(D)** Cocoon width (CW).

The phenotypic data of the 120 samples were screened for outliers, and the results showed that all the data were within a reasonable range (Figure 1). It can be seen that there are significant differences in the four traits among the four breeds of silkworm. The differences provide a basis for the conduction of GWAS.

**FIGURE 2:**
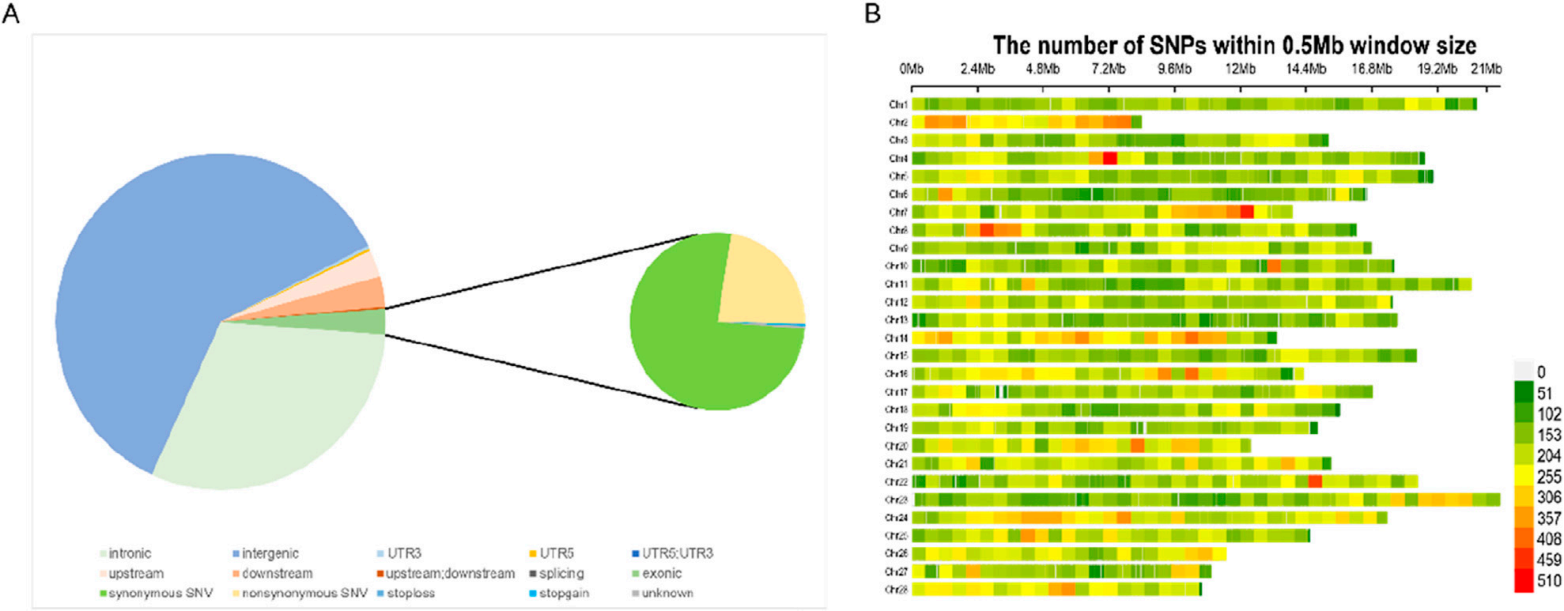
**(A)** Distribution of SNPs identified in four strains within various genomic regions annotated by ANNOVAR. **(B)** The number of SNPs within 0.5 Mb window size in silkworm genome after filtering. Abbreviations: Synonymous SNV (SNPs that do not alter amino acid sequences), upstream/downstream (SNPs within 5 kb upstream or downstream of genes), upstream; downstream (SNPs spanning both upstream and downstream regions), stoploss/stopgain (nonsense SNPs introducing premature stop codons or extending transcripts), *5′UTR/3′UTR* (SNPs in untranslated regions), exonic (coding SNPs), and intronic (non-coding SNPs within introns).

A total of 772.51 Gb of data were obtained after sequencing. After the raw data were filtered out by FASTP (Chen et al., 2018), the clean data of all samples totaled 772.51 Gb, with an average of 6.43 Gb per sample, and the average of the cleanQ30 quality score was 93.98%. The sequencing depth of these samples ranged from 10.79× to 18.7×, with a mean depth of 12.47×. After the calling and filtering, we obtained 6,063,447 SNPs. The resulting SNPs were annotated and it was found that 2.49% of them were located in the exon region (Figure 2A). In the end, we obtained 175,704 SNPs after genotypic filtration and LD pruning. The final SNPs were evenly distributed in the silkworm genome (Figure 2B).

### 3.2 Population stratification analysis

**FIGURE 3:**
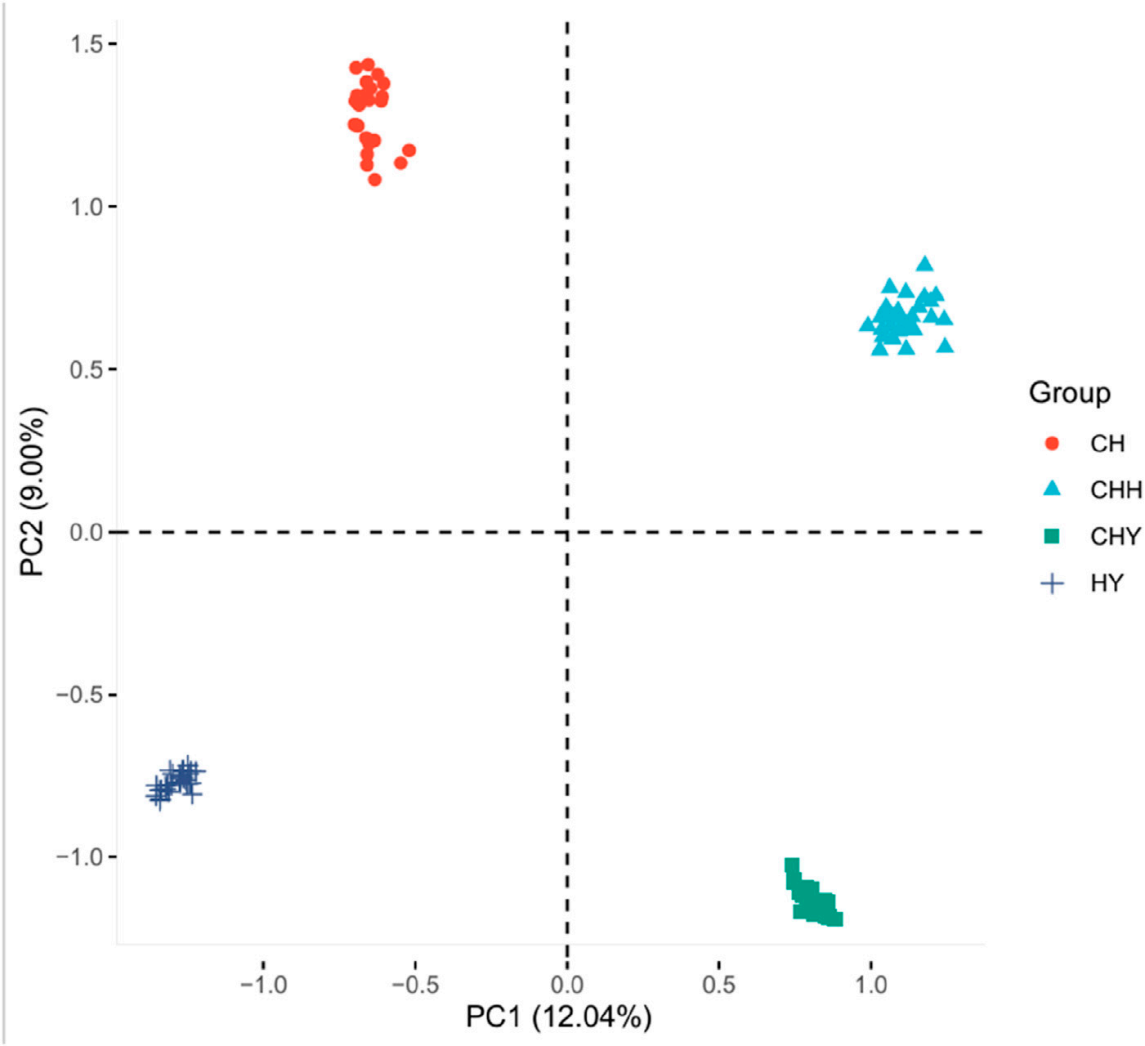
Principal component analysis (PCA) of four Henan silkworm strains. The first three principal components (PC1, PC2, PC3) explain 12.04%, 9.00%, and 7.37% of the total genetic variance, respectively. Each point represents an individual, colored by strain.

PCA results based on genome-wide SNP data showed that population stratification might exist among these silkworms (Figure 3). The first PC explained 12.04% of the variation, and the second PC explained 9.00% of the variation. In addition, we found that the third PC also had a higher percentage, at 7.37%. Therefore, the first three PCs need to be used as covariates in the GWAS model to explain the population stratification effect.

### 3.3 Significant SNPs and candidate genes

**FIGURE 4:**
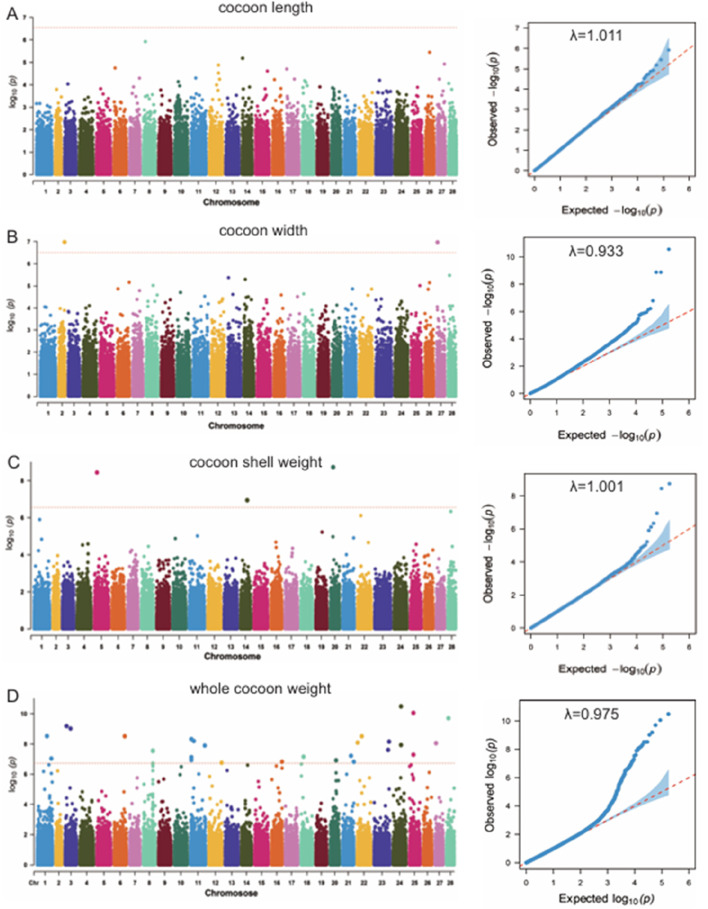
**(A)** Manhattan plot and Q-Q plot of cocoon length (CL) trait. **(B)** Manhattan plot and Q-Q plot of cocoon width (CW) trait. **(C)** Manhattan plot and Q-Q plot of cocoon shell weight (CSW) trait. **(D)** Manhattan plot and Q-Q plot of whole cocoon weight (WCW) trait.

The LMM model was used to analyze the association between the whole genome of the silkworms and the four economic traits, and the relationship between population stratification and individuals was considered. The Manhattan plots and Q-Q plots of the GWAS results based on the LMM are shown in Figure 4. The inflation factors (λ) for the four traits were 1.011, 0.933, 1.001 and 0.975. Based on the results of Q-Q plots and inflation factor, some errors are expected in the model of cocoon width trait, while the model of CSW trait is more accurate than other traits. These results showed that the introduction of the first three principal components as covariates effectively eliminated the effect of population stratification.

The significance threshold obtained by Bonferroni correction is 2.845e-7. Below this threshold, we detected three significant SNPs for the cocoon weight trait, two significant SNPs for the CSW trait, and 35 significant SNPs for the WCW trait (Table 1). By searching within 5 kb upstream and downstream of these significance SNPs, we obtained 28 related genes (Table 1).

**TABLE 1:** Significant SNPs and their information from genome-wide association analysis.

Trait	Chr	Position	P-value	Gene ID	Description
CW	2	7653741	1.04E-07	KWMTBOMO00968	no hit
				KWMTBOMO00969	uncharacterized LOC114250257 isoform X1
	27	1126676	1.07E-07		
				
CSW	5	3377758	2.86E-09	KWMTBOMO02490	ATPase inhibitor-like protein
				KWMTBOMO02491	uncharacterized LOC114241584
	14	8763775	1.18E-07	KWMTBOMO08425	synaptotagmin-10-like isoform X2
	20	3882656	1.48E-09	KWMTBOMO11938	microvitellogenin-like
				KWMTBOMO11939	LOC101744209
				KWMTBOMO11940	microvitellogenin-like
WCW	1	13219760	3.03E-09	KWMTBOMO00047	gamma-soluble NSF attachment protein
	1	15848686	2.39E-07	KWMTBOMO00539	GRB10-interacting GYF protein 2
	1	19161216	9.22E-08	KWMTBOMO00617	protein angel
	3	1854481	6.53E-10	KWMTBOMO01088	fatty-acid amide hydrolase 2-like
	3	7250590	9.59E-10	KWMTBOMO01276	inhibitor of growth protein 3
	6	14325958	3.08E-09	KWMTBOMO03645	LOC114358757
	8	12957991	2.78E-07	KWMTBOMO03581	uncharacterized LOC114253097
	8	12979488	2.89E-08		
	8	12987280	2.30E-07		
	8	12996278	2.74E-08		
	8	13072500	1.85E-07	KWMTBOMO04710	ubiquitin carboxyl-terminal hydrolase 5
	11	1153628	1.15E-07		
	11	1162600	4.78E-09		
	11	1166658	7.50E-08		
	11	4472852	6.28E-09		
	11	19350288	1.27E-08		
	12	17277581	1.75E-07	KWMTBOMO07549	WD repeat-containing protein 26
				KWMTBOMO07550	trafficking protein particle complex subunit 4
	14	8327852	2.53E-07		
	16	14233874	1.51E-07		
	18	365866	2.18E-07	KWMTBOMO10679	hypothetical protein B5V51_11010; partial
	18	3367456	6.95E-08	KWMTBOMO10803	uncharacterized LOC110385585
				KWMTBOMO10804	uncharacterized LOC114252976
	20	8101424	1.21E-07	KWMTBOMO12129	Retrovirus-related Pol polyprotein from transposon TNT 1–94
	21	11446386	6.05E-08	KWMTBOMO12678	OTU domain-containing protein 7B
	21	15275468	1.52E-07		
	22	721868	8.13E-09	KWMTBOMO12824	uncharacterized LOC105842570
	22	6354809	3.10E-09	KWMTBOMO12987	histidine-rich glycoprotein-like
	23	19027987	2.46E-08		
	23	20344373	7.03E-09	KWMTBOMO14200	fatty acyl-CoA reductase wat-like
	24	10672189	1.17E-08		
	24	10672312	3.26E-11		
	25	2533920	2.46E-07		
	25	5634141	5.14E-08	KWMTBOMO15132	zinc finger protein 420 isoform X2
				KWMTBOMO15133	uncharacterized LOC101738733
	25	5641640	8.77E-11		
	27	1361478	8.87E-09		
	28	2794335	1.98E-10		

**FIGURE 5:**
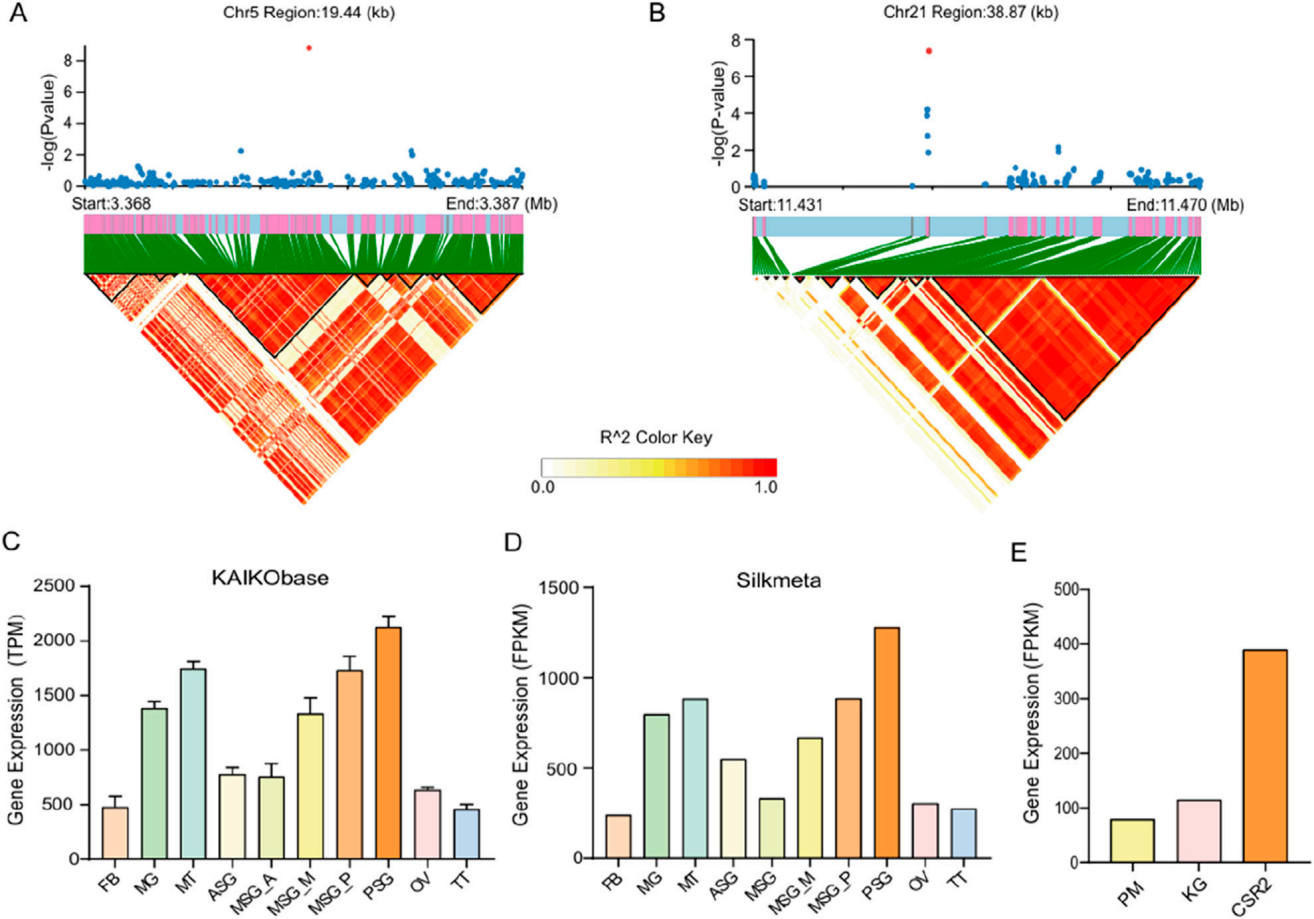
**(A)** LD block of Chr5: 3,360,000–3,380,000 bp region. **(B)** LD block of Chr21: 11,430,000–11,470,000 bp region. Each dot represents a SNP. The green vertical lines and pink blocks denote gene positions. Blocks in the triangle represent pairwise LD (R^2^ values), with darker red indicating higher R^2^ (stronger allelic association). R^2^ ranges from 0 (white) to 1 (red). **(C)** Expression levels of *KWMTBOMO02490* gene in different organs of silkworm (From KAIKObase). **(D)** Expression levels of *KWMTBOMO02490* gene in different organs of silkworm (From Silkmeta). **(E)** Expression of *KWMTBOMO02490* gene in posterior silk glands of three Indian strains (From Silkmeta). (FB: fat body, MG: midgut, MT: Malpighian tubules, ASG: anterior silk glands; MSG: middle silk glands, MSG_A: middle silk glands (anterior), MSG_M: middle silk glands (middle), MSG_P: middle silk glands (posterior), PSG: posterior silk glands, OV: ovary, TT: testis. PM: Pure Mysore, KG: Kolar gold).

In the LD block analysis, we found that there was the high linkage disequilibrium degree near the Chr5: 3377758 and Chr21: 11446386 (overlapping with the GWAS-significant SNPs for CSW and WCW), as shown in Figures 5A,B. It indicates that these two regions were strongly selected in the process of silkworm breeding. These two LD blocks contained the candidate genes *KWMTBOMO02490* (ATPase inhibitor-like protein) and *KWMTBOMO12678* (OTU domain-containing protein 7B).

## 4 Discussion

**FIGURE 6:**
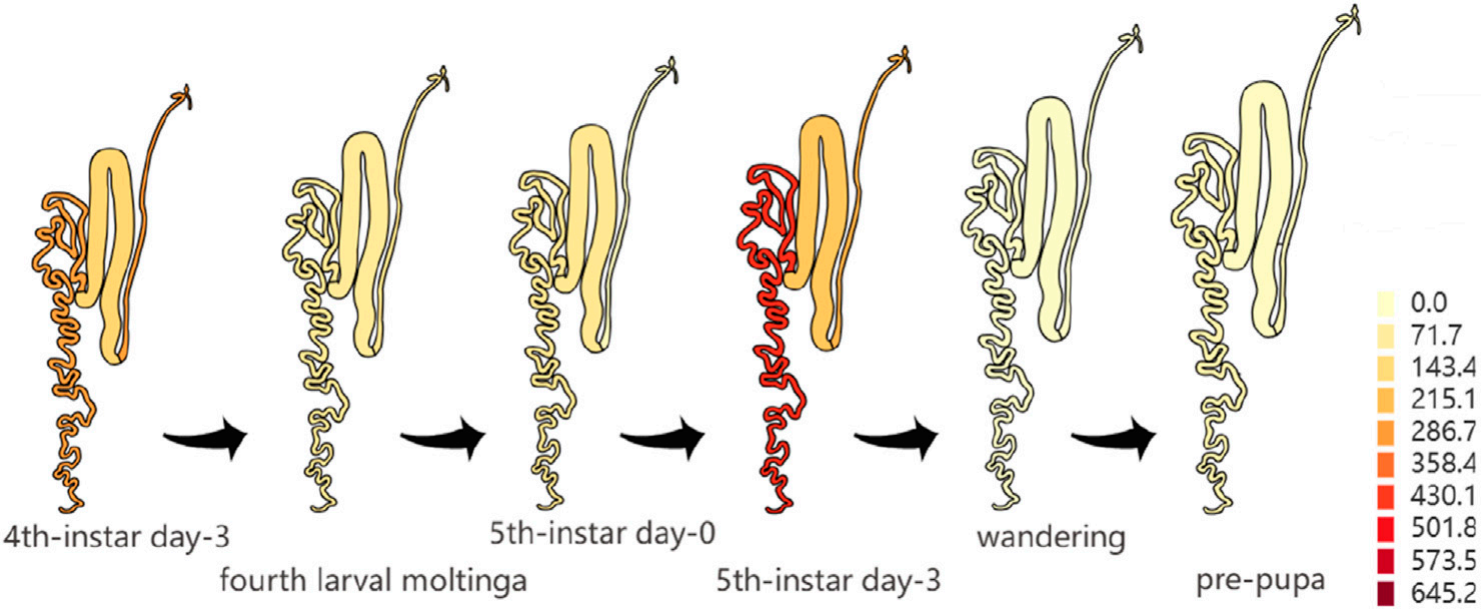
Expression of the *KWMTBOMO02490* gene in silk glands of the domestic silkworm at different periods (Lu F. et al., 2019).

Analyzing the genetic factors of cocoon production is critical for advancing silkworm breeding. GWAS is an important method to identify the genes that regulate economic traits, which can provide new ideas for studying the genetic background of economic traits in silkworms. By GWAS, Tong et al. identified a cluster of tandemly arranged genes coding for sugar transporter proteins (Str) in silkworms, which are synergistically involved in the uptake of flavonoids in silkworms through ‘dose-sharing’ and thus determine the formation of green cocoons (Lu et al., 2023). *KWMTBOMO02490* gene, one of the genes related to the CSW of silkworms in this study, is located in a genome region with a high degree of linkage disequilibrium. It indicated that this gene has been selected with high intensity in the breeding process of domestic silkworms. Notably, we searched three online databases [Silkmeta (Lu et al., 2024), KAIKObase (Shimomura et al., 2009) and SilkDB3.0 (Lu F. et al., 2019)] and found that *KWMTBOMO02490*, has high expression in the posterior silk glands which are the organs that produce fibroin protein (Figures 5C,D). Otherwise, we used transcriptome data of the silk glands from three Indian strains of silkworm in Silkmeta database and found that the expression level of this gene was very different in these three strains. CSR2 (a strain with high CSW) has the highest expression level among these three strains (Figure 5E). Moreover, we found that this gene was also highly expressed in the posterior silk glands of silkworms on the third day of the fifth instar (Figure 6) (Lu F. et al., 2019). These studies suggested that this gene is likely to play an important role in the synthesis of silk. *KWMTBOMO02490* encodes an ATPase inhibitor-like protein, and has ATPase inhibitor activity (GO: 0042030). It is involved in the negative regulation of ATPase activity (GO: 0032780), and locates mainly in mitochondria (GO: 0005739), indicating functions closely related to energy-consuming activities within the cell. The process of fibroin protein synthesis in the posterior silk gland requires a large amount of energy, which comes from ATP hydrolysis. In summary, we speculate that ATPase inhibitor-like proteins affect the synthesis of silk fibroin in the posterior silk glands of silkworms by regulating the activity of ATPase, thereby affecting silk yield. For the WCW-related genes, we found that the region of the *KWMTBOMO12678* gene also showed a high degree of linkage disequilibrium. This gene encodes OTU domain-containing protein 7B (OTUD7B), which is a deubiquitylase belonging to the A20 subgroup of ovarian tumor (OTU) protein superfamily (Tang et al., 2021). A previous study has shown that the OTUD7B deficiency results in B-cell hyper-responsiveness to antigens, lymphoid follicular hyperplasia in the intestinal mucosa, and elevated host-defence ability against an intestinal bacterial pathogen, *Citrobacter rodentium* (Hu et al., 2013).

## 5 Conclusion

In this study, GWAS was used to study the cocoon production traits of four silkworm varieties in Henan Province, and 40 significant SNPs with 28 related candidate genes were identified. These genes are closely related to traits such as CSW, CW, and full cocoon weight. The results suggest that these candidate genes may affect the production and health traits of silkworms, and in particular, *KWMTBOMO02490* gene is considered to be an excellent candidate genes affecting cocoon shell traits due to its potential role in the posterior silk glands. These findings not only enhance our understanding of the genetic basis of cocoon production traits, but also provide a new perspective on molecular marker-assisted selection of silkworm varieties.

Although the identified candidate genes may be related to cocoon production traits, their specific biological functions and mechanisms of action need to be further experimentally verified. Future research will focus on the functional validation and application of these candidate genes, with a view to breeding new silkworm varieties with higher cocoon yields.

## Data Availability

The datasets presented in this study can be found in online repositories. The names of the repository/repositories and accession number(s) can be found in the article/supplementary material.
